# Non-targeted urinary metabolomics in pregnancy and associations with fetal growth restriction

**DOI:** 10.1038/s41598-020-62131-7

**Published:** 2020-03-24

**Authors:** Chelsea M. Clinton, James R. Bain, Michael J. Muehlbauer, YuanYuan Li, Leping Li, Sara K. O’Neal, Brenna L. Hughes, David E. Cantonwine, Thomas F. Mcelrath, Kelly K. Ferguson

**Affiliations:** 1 Division of Maternal Fetal Medicine, Department of Obstetrics and Gynecology, Duke University Medical Center, Durham, NC USA; 2 Metabolomics Laboratory, Duke Molecular Physiology Institute, and Division of Endocrinology, Metabolism, and Nutrition, Department of Medicine, Duke University School of Medicine, Durham, NC USA; 3 Duke Molecular Physiology Institute, Sarah W. Stedman Nutrition and Metabolism Center, Duke University Medical Center, Durham, NC USA; 4 Biostatistics and Computational Biology Branch, Intramural Research Program, National Institute of Environmental Health Sciences, Research, Triangle Park, NC USA; 5 Division of Maternal-Fetal Medicine, Brigham & Women’s Hospital, Harvard Medical School, Boston, MA USA; 6 Epidemiology Branch, Intramural Research Program, National Institute of Environmental Health Sciences, Research, Triangle Park, NC USA

**Keywords:** Metabolomics, Predictive markers

## Abstract

Our objective was to identify metabolites associated with fetal growth restriction (FGR) by examining early and late pregnancy differences in non-targeted urinary metabolites among FGR cases and non-FGR controls. An exploratory case-control study within LIFECODES birth cohort was performed. FGR cases (N = 30), defined as birthweight below the 10^th^ percentile, were matched with controls (N = 30) based on maternal age, race, pre-pregnancy body mass index, and gestational age at delivery. Gas chromatography/electron-ionization mass spectrometry was performed on urine samples collected at 10 and 26 weeks of gestation. Differences in urinary metabolite levels in cases and controls at each time point and between the two time points were calculated and then changes compared across pregnancy. 137 unique urinary metabolites were annotated, and several identified that were higher in cases compared to controls. For example, urinary concentrations of benzoic acid were higher in cases compared to controls at both study visits (3.01-fold higher in cases at visit 1, p < 0.01; 3.10-fold higher in cases at visit 3, p = 0.05). However, these findings from our exploratory analysis were not robust to false-discovery-rate adjustment. In conclusion, using a high-resolution, non-targeted approach, we found specific urinary organic acids differed over pregnancy by FGR case status.

## Introduction

Fetal growth restriction (FGR) is classically defined by an estimated fetal weight of less than the 10^th^ percentile. It is a serious complication of pregnancy that is associated with adverse perinatal outcomes, including intrauterine demise, neonatal morbidity, and neonatal death^1^. It has also been associated with an increased risk of developing chronic metabolic diseases in adulthood^2^. Some risk factors for FGR include maternal cigarette smoking in pregnancy, hypertensive disease, and infection; however, prenatal identification of cases still relies on ultrasound measurements which have poor prognostic ability^3^. In the case of idiopathic FGR without obvious underlying risk factors necessitating serial growth ultrasounds, this diagnosis could be delayed or missed.

The advent of metabolomics techniques for capturing markers in the onset of disease offers promise for prediction of multiple adverse pregnancy outcomes, including FGR. Nuclear magnetic resonance (NMR) spectroscopy, and gas chromatography/mass spectrometry (GC/MS), and liquid chromatography/MS metabolomics approaches have the capacity to quantify biomarkers of metabolic responses of living systems to disease, toxins, or nutritional factors in biofluids and other biological samples. Clinical literature has highlighted the utility of metabolomics for prognosis of pregnancy complications, such as gestational diabetes and preeclampsia^4,5^, but the  application to studies of FGR, despite showing promise, has been more limited^6–9^. Particularly, no study to date has employed GC/MS to interrogate urinary metabolites that are associated with fetal growth.

In the present study, we sought to examine the utility of prenatal urinary metabolites in classification of pregnancies complicated by FGR. We expand upon previous work by adhering to a stricter definition of idiopathic FGR, defined as birth weight <10^th^ percentile for gestational age in pregnancies uncomplicated by comorbidities such as preeclampsia. We also examined metabolites in urine samples collected at two time points per participant in pregnancy, and performed measurements using high-resolution GC/MS which has not been utilized previously in a study of metabolomics and fetal growth. Our objectives were to identify metabolites, or changes in metabolites during pregnancy, that are associated with FGR and to test the ability of a classification and regression tree (CART) to distinguish cases of FGR from controls using these biomarkers.

## Results

Table 1 presents the participants’ demographic, behavioral, and pregnancy-related characteristics. Matched and unmatched characteristics were similar in case and control groups. The mean birthweight of neonates in the control group was 3.2 kg compared to 2.2 kg among cases. This corresponded to a mean percentile of 51.5 in controls (range 11–98) and a mean percentile of 3.0 in cases (range 0–6.2).

**Table 1:** Population demographic and pregnancy characteristics by case-control status: Mean (standard deviation) or n (%).

	Cases (N = 30)	Control (N = 30)
**Matched variables**		
Age (years)	32.3 (6.4)	32.9 (5.4)
Race/ethnicity		
White	18 (60.0)	18 (60.0)
Black	6 (20.0)	6 (20.0)
Other (Asian, Hispanic)	6 (20.0)	6 (20.0)
Pre-pregnancy body mass index (kg/m^2^)	24.5 (5.9)	25.4 (5.8)
Gestational age at delivery (weeks)	38.0 (0.9)	38.5 (1.1)
**Non-matched variables**		
Gravidity		
Missing	1	0
0–1 previous pregnancy	14 (48.3)	17 (56.7)
>1 previous pregnancy	15 (51.7)	13 (43.3)
Parity		
Missing	1	0
Nulliparous	7 (24.1)	10 (33.3)
Parous	22 (75.9)	20 (66.7)
Health insurance provider		
Private	21 (70.0)	25 (83.3)
Public	7 (23.3)	5 (16.7)
No insurance	2 (6.7)	0
Education level		
Missing	0	1
Junior college/some college and below	11 (37)	8 (27.5)
College graduate	12 (40)	12 (41.4)
Some graduate school, professional school, or above	7 (23.3)	9 (31)
Alcohol use during pregnancy		
None	28 (93.3)	29 (96.7)
Some	2 (6.7)	1 (3.3)
Tobacco use during pregnancy		
None	26 (86.7)	29 (96.7)
Some	4 (13.3)	1 (3.3)
Visit 1 gestational age (weeks)	10.8 (2.3)	10.9 (2.1)
Visit 3 gestational age (weeks)	25.5 (1.1)	25.5 (0.9)
Birthweight (kg)	2.2 (0.3)	3.2 (0.5)

We identified 397 unique spectral peaks and annotated 204 metabolites in the urine samples analyzed. Of these, 137 metabolites were detected in over 50% of samples. The names of these metabolites as well as the percent detection by case status and visit are shown in Supplemental Table 1. Fold changes in urinary metabolites that differed significantly (p < 0.05) between cases and controls at either Visit 1 or Visit 3 are presented in Table 2. At Visit 1, the greatest fold changes between cases and controls were observed for 2-methylglutaric acid (4.14-fold higher in cases compared to controls, p = 0.03) and acetoacetate (5.35-fold higher in cases compared to controls, p = 0.04). At Visit 3, the greatest fold change was observed for 1,2-propanediol (3.76-fold higher in cases compared to controls, p = 0.01). The only metabolite that was consistently different at both visits was benzoic acid, which was approximately 3-fold higher in cases compared to controls at both visits.

**Table 2:** Mean fold change (p values) in urinary metabolites, with p < 0.05 at either Visit 1 or Visit 3, in cases of fetal growth restriction compared to controls.

Annotation		Visit 1		Visit 3	
	CAS Registry Number	Fold change	p value	Fold change	p value
1,2-Propanediol	57-55-6	1.95	0.86	3.76	0.01
Kynurenic acid	492-27-3	2.51	0.34	1.39	0.03
n-Heptanoic acid	111-14-8	2.30	0.17	2.79	0.04
Benzoic acid	65-85-0	3.01	<0.01	3.10	0.05
Malonic acid	141-82-2	2.73	0.02	2.43	0.12
2-Ketoleucine/ketoisoleucine	816-66-0	3.03	0.03	2.50	0.26
2-Ketobutyric acid	600-18-0	3.01	0.02	1.88	0.64
2-Methylglutaric acid	617-62-9	4.14	0.03	1.84	0.66
Acetoacetate	541-50-4	5.35	0.04	2.01	0.99

Note: Results presented for metabolites that were significantly (p < 0.05) different in cases compared to controls at one or both study visits as calculated by paired t-test. No associations were significant after FDR adjustment (all q > 0.05). Abbreviations: CAS, Chemical Abstracts Service.

We also noted several differences in how urinary metabolite concentrations changed over pregnancy between cases and controls (Table 3). For example, 2-methylglutaric acid levels only increased 1.52-fold from Visit 1 to Visit 3 in cases of FGR but levels increased 2.73-fold in controls (p for difference in change <0.01). For cholesterol, levels increased from Visit 1 to Visit 3 in cases (2.48-fold change) but not as much as they did in controls (6.54-fold change, p for difference in change=0.02). The statistical significance of these associations was not robust to false discovery rate adjustment (q > 0.05).

**Table 3:** Mean fold change in urinary metabolites from Visit 1 to Visit 3 in cases and controls with p < 0.05.

Annotation	CAS Registry Number	Change in Cases	Change in Controls	p-value
2-Methylglutaric acid	617-62-9	1.52	2.73	<0.01
Cholesterol	57-88-5	2.48	6.54	0.02
Urocanic acid	104-98-3	1.26	2.33	0.02
2-Phenylacetamide	103-81-1	3.92	2.04	0.02
2-Ketobutyric acid	600-18-0	1.53	2.11	0.02
Glycine	56-40-6	3.05	1.60	0.03
Kynurenic acid	492-27-3	1.20	1.61	0.03
3-(3-Hydroxyphenyl)propionic acid	621-54-5	2.51	7.41	0.05

Note: p-value corresponds to paired t-test comparing changes in cases and controls. Results presented for metabolites with significant (p < 0.05) difference in changes between Visit 1 and Visit 3 in cases compared to controls. No associations were significant after FDR adjustment (all q > 0.05). Abbreviations: CAS, Chemical Abstracts Service.

**Figure 1:**
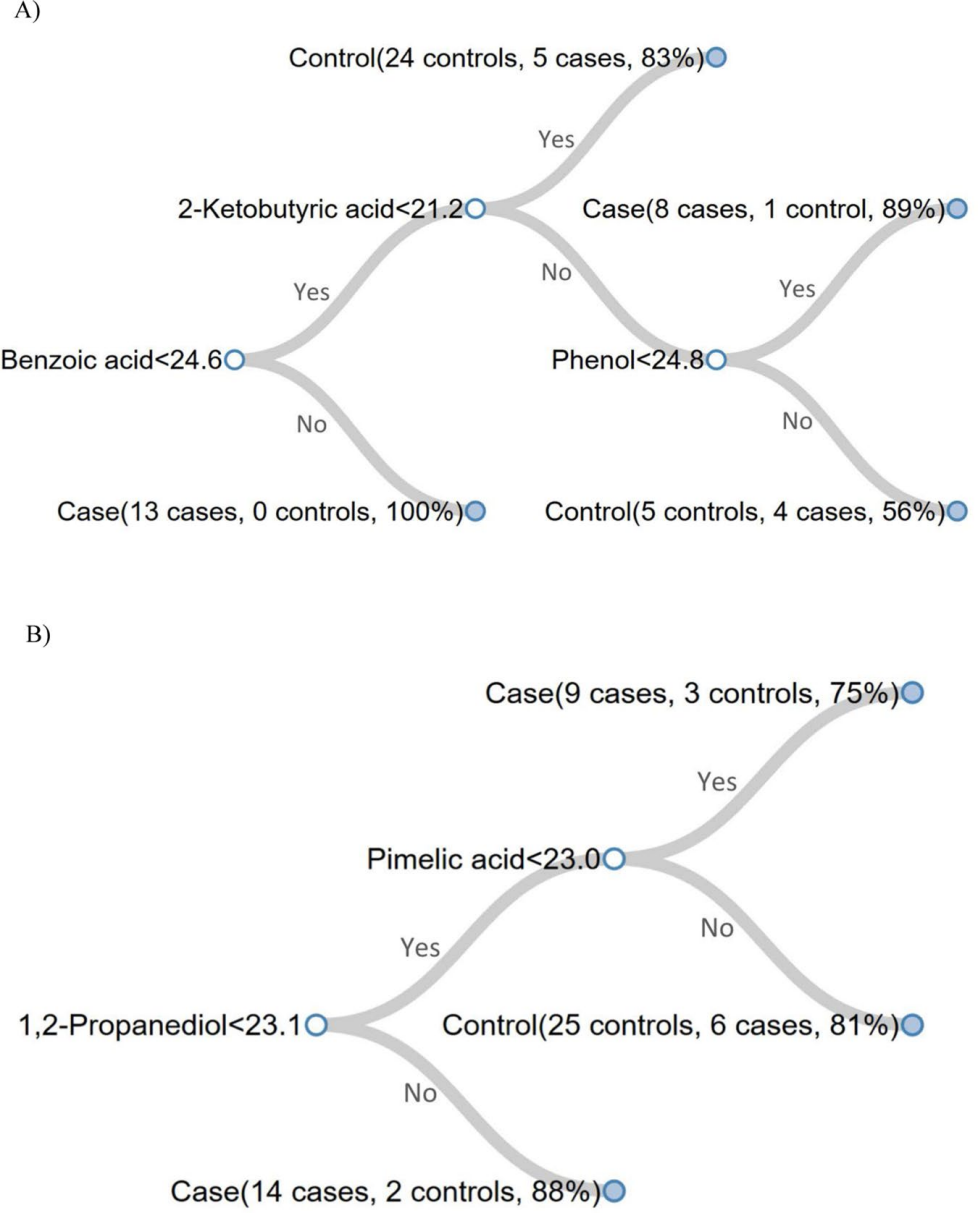
Classification trees obtained using all samples for Visit 1 (**A**) Visit 3 (**B**). In a tree, unfilled circles represent nodes and solid circles represent terminal leaves. Each node shows the name of the metabolite and the rule for splitting. Each leaf, which is termed “case” or “control” based on the rule, lists the number of cases and controls that were classified to the leaf in the CART procedure and the corresponding posterior probability.

In our CART analysis, we observed good classification accuracies of FGR cases using the urinary metabolites measured (83.3% for Visit 1 and 81.4% for Visit 3). Similarly, sensitivity (96.7% for Visit 1 and 83.3% for Visit 3) and specificity (70% for Visit 1 and 79.3% for Visit 3) were good. Classification trees for Visit 1 and Visit 3 are displayed in Fig. 1. Unfilled circles represent nodes, which indicate the name of the metabolite that creates the split as well as the rule (*i.e*., cutoff) for splitting. Solid circles represent terminal leaves and show the number of cases and controls classified to the leaf, as well as the corresponding posterior probability of case/control assignment to that leaf.

The tree can be interpreted as follows. For Visit 1, if the participant’s urinary benzoic acid level is ≥24.6 units, then the outcome is FGR (i.e., “case”) with a posterior probability of 100% (13 cases, 0 controls). If the participant’s benzoic acid level is <24.6 and their 2-ketobutyric acid level is ≥21.2 units and their phenol level is <24.8, then the outcome is FGR (i.e., “case”) with a posterior probability of 89% (8 cases, 1 control), and so on. For Visit 3, if the participant’s 1,2-propanediol level is ≥23.1 units, the outcome is FGR with a posterior probability of 88% (14 cases, 2 controls). If the participant’s 1,2-propanediol level is <23.12 and their pimelic acid level is <23.0 units, then the outcome is FGR (i.e., “case”) with a posterior probability of 75% (9 cases and 3 controls). The first nodes described have the greatest importance for classification, as in all CART models.

In our test of the robustness of these results, we observed that the average training and testing accuracies were 83.1% and 73.3%, respectively, for Visit 1, and 81.4% and 76.3%, respectively, for Visit 3. In the permutation study, where we tested whether it was likely that the results observed could be due to chance, the mean training accuracy was still 83%. The mean testing accuracy, i.e., the mean prediction accuracy for the imputed data, was much lower (49.1%, equivalent to chance) than what we observed in the observed data, with only 527 of the 10,000 permutations resulted in a testing accuracy equal to or higher than that for the observed data. This indicates that the classification results obtained from the observed data are meaningful and the good performance achieved is unlikely due to chance.

## Discussion

In this study of fetal growth restriction, we identified maternal urinary metabolites from early as well as mid pregnancy that were different in cases compared to controls at nominal statistical significance. Benzoic acid was higher among FGR cases at both visits. We additionally observed that there were patterns of change in some metabolites across gestation that differed between cases and controls. Finally, despite the small sample size in our study, we demonstrated that combinations of urinary metabolites were useful for classifying cases of FGR. The results from this exploratory, untargeted analysis may identify important physiological (i.e., endogenous) factors that are pertinent to the origin and development of growth restriction in pregnancy that deserve additional targeted attention in future work.

Previous studies have examined a similar research question but have not been consistent. This could be due to the use of NMR spectroscopy methods, differences in outcome definition, or to uncontrolled urine dilution. In a 2014 study, non-targeted urinary metabolites were analyzed in samples from early pregnancy (11–13 weeks of gestation; N = 438)^7^. Four out of 34 urinary metabolites were associated with FGR, defined as birthweight <10^th^ percentile, including acetic acid, formic acid, tyrosine and trimethylamine. Luthra *et al*. were unable to replicate these findings in a nested case-control study of FGR (n = 53 cases, n = 106 controls)^6^. In a third study, urine samples collected from both the first and third trimesters from two independent birth cohorts in Spain were again analyzed by NMR^8^. They identified 10 metabolites from the third trimester that were associated with estimated fetal weight, birthweight, and placental weight that were reproducible across the two birth cohorts; however, the findings from the other two studies were not replicated.

Our study had three major differences from this previous work that may make our findings more replicable in future. First, we had strict inclusion criteria for FGR cases. Other cutoffs could results in a very heterogeneous mix of individuals among cases with very different metabolic profiles. Improved case definition could enhance consistency across analyses in this area and make these markers more useful for eventual prediction. Second, we used GC/MS for non-targeted metabolomics. NMR spectroscopy has the ability to detect very small metabolites but with less sensitivity than GC/MS^10^. Using the more sensitive approach could enhance replicability as well. Finally, we adjusted for urine dilution by analyzing samples that were equivalent in creatinine concentrations. Failing to adjust for urine dilution would results in metabolite differences that can be attributable to hydration status or other clearance parameters that would obscure real differences in metabolites in cases of FGR compared to controls. These study features should be incorporated into future work to improve replication across studies and increase the likelihood that these markers could eventually be used for early diagnosis of FGR.

Clinically significant differences in urinary metabolites were primarily related to human or gut-microbial metabolism of essential (ES) or conditionally essential (CES) amino acids (AA), including urocanic acid (from histidine, ES), 2-phenylacetamide (from phenylalanine, ES), 2-ketobutyric acid (from methionine or threonine, both ES), glycine (itself a CES), kynurenic acid (from tryptophan, ES), and 3-(3-hydroxphenyl) propionic acid (from tyrosine, CES or phenylalanine, ES). This suggests that FGR might be related to broad-scale derangements in metabolism of essential AA, either through the mother’s ES-AA nutritional exposures, her intrinsic ES-AA metabolism, her gut-microbial metabolism of ES-AA, placental transfer, or placental or fetal catabolism of these key macronutrients. It is known that the fetoplacental unit makes heavy use of amino acids, and maternal metabolism must adapt to supply the fetus with required nutrients. Amino acids are also highly dependent on active transport processes^11^ that are influenced by morphological characteristics of the placenta, such as size and vascularity^11,12^. Since FGR is often a consequence of placental insufficiency^13^, characterized by decreased placental volume and increased resistance to blood flow, this could explain some of the changes observed here.

Metabolomic analyses of cord blood from FGR pregnancies have revealed significant differences in essential amino acids as well as abnormal lipid metabolism, consistent with the findings from our analysis^14^. We observed that cholesterol increased only slightly over pregnancy in cases (2.48-fold change) but levels increased much more dramatically in controls (6.54-fold change). A study of NMR-based metabolic profiling of maternal and cord blood plasma samples revealed that both the mother and fetuses of pregnancies complicated with FGR have substantial disruptions in lipid metabolism^15^.

Another metabolite with potential biological significance in the development of FGR identified in our study is benzoic acid, which was approximately 3-fold higher in cases compared to controls at both Visit 1 and Visit 3, and which was identified as an important predictor in our classification and regression tree from Visit 1. Benzoic acid, found in plant-based foods and used as a food additive, is also a product of gut-microbial catabolism of phenylalanine (ES-AA), tyrosine (CES-AA), or botanical polyphenols in the diet^16^. Catabolism of phenylalanine and tyrosine can yield benzoic acid, which can undergo hepatic or renal phase II conjugation with glycine. This glycine conjugate of benzoic acid, called hippuric acid, can then be excreted in the urine. Hippuric acid has been found to be elevated in the circulation of patients with uremia^17–19^. In the present study, benzoic acid and hippuric acid levels increased with time in both cases and controls. Free benzoic acid was higher in cases at both time points, suggesting heavier benzoate loading (e.g., ingestion) or impaired hepatic or renal detoxification of benzoate to hippuric acid in the cases. Additionally, due to the fact that gut microbes produce benzoic acid by their actions on foodstuffs and small-bowel transit is slowed during pregnancy^20^, this may also affect levels of benzoic acid.

CART analysis showed that combinations of only a small number of metabolites could classify individuals based on case status in our study population. At Visit 1, benzoic acid and 2-ketobutyric acid had the greatest importance and at Visit 3 1,2-propanediol and pimelic acid were the only necessary predictors. The testing accuracies of 73% and 76% for Visits 1 and 3, respectively, are quite high compared to the testing accuracy in the permutation study (49.1%) which evaluates what happens under chance after shuffling of the values (i.e., when case assignment is random). Furthermore, the high training accuracy observed in the permutation study (83%) and in the observed data (81–83%) indicates that the trees generated using the real data were not random (i.e., are learning appropriately). Overall this suggests that metabolites were useful for classifying cases of FGR, and future work in prospective cohort studies could utilize metabolomics in combination with CART to develop predictive models.

Our study had several strengths, including our strict inclusion criterion to attempt to identify cases of idiopathic FGR. This allowed us to investigate metabolites that were independent of factors commonly associated with disorders of fetal growth, including maternal obesity and diabetes, fetal anomalies, and preeclampsia. In addition, we focused our analysis on the smallest babies in the LIFECODES birth cohort, with almost all falling below the 5^th^ percentile for birthweight at delivery. This likely improved our identification of true FGR newborns rather than those that are constitutionally small. Finally, we applied CART analysis, which showed how multiple metabolites could be useful for classifying cases of FGR.

A weakness of our study is our small sample size, which could have limited our power to detect true differences in metabolite concentrations in cases compared to controls (type II error). Furthermore, because we had a large number of comparisons and our results were not robust to FDR adjustment, our primary findings could be attributed to chance (type I error). However, our results demonstrate promise for the use of GC/MS metabolomics in the study of FGR, and how assessing urinary metabolites at multiple time points in pregnancy may be informative, since the differences we observed between cases and controls were not entirely consistent at our two study visits. This method and the repeated-sampling strategy should be applied to larger studies on this topic. Another limitation is our use of a matched design. While this may have helped to reduce variability due to the matching factors, such as maternal BMI, this could limit the clinical use, replicability, and interpretation of our findings. Future work should use an unmatched design to better understand the influence of covariates on the associations between urinary metabolites and FGR and to develop prediction models. Finally, our study was limited by lack of information on genetic, environmental, occupational, dietary, and other lifestyle factors. Information on these predictors would be important for contextualizing our findings for the understanding of modifiable factors that could be driving the observed associations between urinary metabolites and FGR. Capturing this information should also be a priority in future work.

Our exploratory study of prenatal urinary metabolites in mothers of FGR babies as well as controls showed several differences in metabolites that reflect essential amino acid metabolism and catabolism in the two groups. These differences are dependent on timing of measurement. In addition to comparing concentrations at multiple time points, examining differences in how metabolite concentrations change across pregnancy may be valuable. Application of non-targeted metabolomics using GC/MS should be applied to larger longitudinal studies of pregnancy in order to establish mechanisms and/or predictive biomarkers for growth restriction.

## Methods

LIFECODES is an ongoing, prospective birth cohort begun in 2006 at Brigham and Women’s Hospital (BWH) in Boston, MA. The purpose of the study is to examine environmental and behavioral factors associated with adverse outcomes of pregnancy. Women are recruited early in gestation (prior to 15 weeks) at BWH or affiliated academic practices and are included in the study if they are carrying a non-anomalous fetus and plan to deliver at BWH. At the first study visit (visit 1, targeted at 10 weeks of gestation), the mother provides informed consent and is asked to provide spot urine and blood samples, as well as questionnaire information pertaining to demographic characteristics and medical history. At subsequent visits 2–4, targeted at 18, 26, and 35 weeks gestation, she provides additional spot urine and blood samples. Gestational age is estimated according to American College of Obstetricians and Gynecologists (ACOG) recommendations^21^. At delivery, detailed information on any complications as well as gestational age and birthweight are recorded. The Institutional Review Board at Brigham and Women’s Hospital approved this study protocol. The use of data and biological specimens for the present project was deemed exempt by the National Institute of Environmental Health Sciences, National Institutes of Health. All methods were performed in accordance with relevant guidelines.

For the present exploratory case-control study, our objective was to identify cases of idiopathic FGR and compare them to controls. We thus excluded pregnancies that were complicated by multiple gestation, pre-eclampsia, congenital or chromosomal anomalies, or pre-existing diabetes (type I or II) from selection. We then selected from the LIFECODES birth cohort 30 term (>37 weeks gestation) singleton cases of FGR, defined as birthweight below the 10^th^ percentile for gestational age, based on the criteria published by Oken *et al*.^22^, with a preference for cases born below the 5^th^ percentile for gestational age. In other words, we tried to select the smallest babies for examination in this study since our sample size was small. Controls from the same underlying population were 1:1 matched to the cases, based on the following criteria: maternal age (±5 years); maternal race (White, African American, Other); maternal pre-pregnancy body mass index (BMI;±5 kg/m^2^); and gestational age at delivery (± 2 weeks). Women were not matched by health insurance provider, an indicator of socioeconomic status. We also did not match on gestational age at the time of urine sample collection because most samples were collected with a narrow (2 week) window.

### Urinary metabolomics by mass spectrometry

For analysis, we selected previously unthawed urine aliquots from visits 1 and 3 that were stored at − 80 °C. One aliquot was missing for a case participant at visit 3, but otherwise all participants had urine samples available at both study visits (n = 60 samples at visit 1; n = 59 samples at visit 3). We were unable to analyze all urine samples collected during pregnancy due to cost constraints, and thus selected samples from early as well as mid pregnancy as key periods for the development of FGR. Urine samples underwent non-targeted metabolomics via gas chromatography/electron-ionization mass spectrometry (GC/EI-MS) in the Metabolomics Laboratory of the Duke Molecular Physiology Institute (DMPI), as described by McNulty *et al*.^23^. Briefly, urinary creatinine was measured by the Jaffe method using a DxC 600 clinical analyzer and reagents from Beckman (Brea, CA). Using a volume of urine equivalent to two micromoles of creatinine, small metabolites were extracted twice into ethyl acetate, dried, methoximated, derivatized with *N*-methyl-*N*-trimethylsilyl-trifluoroacetamide (MSTFA), and run on an 6890 N GC-5975 Inert MS (Agilent Corporation, Santa Clara, CA), with the MS set to scan broadly from *m/z* 50 to 600 during a GC heat ramp from 60° to 325 °C. Raw data (integrated peak areas) from Agilent’s ChemStation software environment were imported into the freeware, Automatic Mass Spectral Deconvolution and Identification Software or AMDIS, provided courtesy of the National Institute of Standards and Technology or NIST, at http://chemdata.nist.gov/mass-spc/amdis/^24^ Deconvoluted spectra were annotated as metabolites, to the extent possible, using an orthogonal approach that incorporates both retention time (RT) from GC and the fragmentation pattern observed in MS. Peak annotation was based primarily on DMPI’s own RT-locked spectral library of human metabolites, which is now one of the largest of its kind for GC/EI-MS (2039 spectra from 1165 unique compounds, as of March 2019). This library is built upon the Fiehn GC/MS Metabolomics RTL Library (a gift from Agilent, their part number G1676AA)^25^. Additional spectra for comparison have been gleaned from the Golm Metabolome Library (courtesy of Dr. Joachim Kopka and coworkers at the Max Planck Institute of Molecular Plant Physiology, Golm, Germany; http://csbdb.mpimp-golm.mpg.de/csbdb/gmd/gmd.html)^26^ and other public spectral libraries, and by acquiring or synthesizing pure reagent standards and running them.

To limit the number of comparisons, and for improved interpretation, we restricted our analysis to annotated metabolites. All metabolite peak areas were log-base-2 transformed to approximate normality, because the distributions of individual metabolites were highly skewed. Concentrations below the limit of detection for each metabolite were imputed with the minimum value observed across the dataset. Metabolites with greater than 50% below the limit of detection were excluded from analysis.

### Statistical analysis

Our primary outcome was fold change in urinary metabolites in cases of FGR compared to controls. We calculated the log_2_ fold change for each metabolite in cases compared to controls by taking the difference in log_2_-transformed mean metabolite levels, which would be equivalent to taking the ratio of two concentrations on the untransformed scale. We then converted these values to the raw fold change by exponentiating 2 to the value of the difference. We tested for statistical differences using a paired t-test of log_2_-transformed values to account for matching. Additionally, we sought to examine whether fold change in each metabolite from visit 1 to visit 3 was different in cases compared to controls. To examine these differences, we calculated fold change from visit 1 to visit 3 within each group and then tested the differences in these values using a paired t-test. Fold change over time was calculated in the same manner as fold change between groups. Due to the exploratory nature of our study, we presented p values from statistical tests and highlighted findings that were consistent across visits or that had clinical significance (i.e., large effect sizes). However, we additionally calculated q values using the Benjamini Hochberg procedure to examine the effect of controlling for false discovery rate.

Our second objective was to utilize the annotated metabolites to develop a classification model for FGR. For this analysis we used the CART method, which first uses binary splitting to divide the feature space, defined by the values of the metabolites, into non-overlapping regions, and, second, fits a regression model for each region to classify the outcome for samples in that region^27^. The splitting process is performed recursively, and at each stage the split point is chosen to optimize an objective function, typically a loss function. The process results in a single tree-like structure that best classifies the response variable based on the predictor variables in a dataset. We used fitctree, a binary classification decision tree implemented in Matlab®, as the classification method^28^. To test the robustness of our results, we used leave-one-out cross-validation to assess model over-fitting, and we performed a permutation analysis to evaluate the probability that classifications could have occurred by chance. Additional details of the CART methods are described in the Supplementary Material.

## Supplementary information

Supplementary Materials.

## References

[1] Resnik R (2002). Intrauterine growth restriction. Obstet. Gynecol..

[2] Barker DJ (2006). Adult consequences of fetal growth restriction. Clin. Obstet. Gynecol..

[3] American College of Obstetricians and Gynecologists (2013). Practice Bulletin No. 134: Fetal growth restriction. Obstet. Gynecol..

[4] Nobakht M (2018). Gh. Bf. Application of metabolomics to preeclampsia diagnosis. Syst. Biol Reprod. Med..

[5] Chen Q, Francis E, Hu G, Chen L (2018). Metabolomic profiling of women with gestational diabetes mellitus and their offspring: Review of metabolomics studies. J. Diabetes Complications.

[6] Luthra G (2018). First and second trimester urinary metabolic profiles and fetal growth restriction: an exploratory nested case-control study within the infant development and environment study. BMC pregnancy and childbirth.

[7] Maitre L (2014). Urinary metabolic profiles in early pregnancy are associated with preterm birth and fetal growth restriction in the Rhea mother-child cohort study. BMC medicine.

[8] Maitre L (2016). Maternal urinary metabolic signatures of fetal growth and associated clinical and environmental factors in the INMA study. BMC medicine.

[9] Breslau N, Paneth N, Lucia VC, Paneth-Pollak R (2005). Maternal smoking during pregnancy and offspring IQ. *Int*. J. Epidemiology.

[10] Pan Z, Raftery D (2007). Comparing and combining NMR spectroscopy and mass spectrometry in metabolomics. Anal. Bioanal. Chem..

[11] Lager S, Powell TL (2012). Regulation of nutrient transport across the placenta. J Pregnancy.

[12] Fowden AL, Ward JW, Wooding FP, Forhead AJ, Constancia M (2006). Programming placental nutrient transport capacity. J. Physiol..

[13] Zhang S (2015). Placental adaptations in growth restriction. Nutrients.

[14] Sanz-Cortes M (2013). Metabolomic profile of umbilical cord blood plasma from early and late intrauterine growth restricted (IUGR) neonates with and without signs of brain vasodilation. PLoS One.

[15] Miranda J (2018). Metabolic profiling and targeted lipidomics reveals a disturbed lipid profile in mothers and fetuses with intrauterine growth restriction. Sci. Rep..

[16] Penczynski KJ (2017). Relative validation of 24-h urinary hippuric acid excretion as a biomarker for dietary flavonoid intake from fruit and vegetables in healthy adolescents. Eur. J. Nutr..

[17] Zhao YY (2013). Metabolomics in chronic kidney disease. Clin. Chim. Acta.

[18] Niwa T (2011). Update of uremic toxin research by mass spectrometry. Mass. Spectrom. Rev..

[19] Toyohara T (2010). Metabolomic profiling of uremic solutes in CKD patients. Hypertens. Res..

[20] Everson GT (1992). Gastrointestinal motility in pregnancy. Gastroenterol. Clin. North. Am..

[21] Committee opinion no 611: method for estimating due date. *Obstet. Gynecol*. **124**, 863–866 (2014). 10.1097/01.AOG.0000454932.15177.be 25244460

[22] Oken E, Kleinman KP, Rich-Edwards J, Gillman MW (2003). A nearly continuous measure of birth weight for gestational age using a United States national reference. BMC pediatrics.

[23] McNulty NP (2011). The impact of a consortium of fermented milk strains on the gut microbiome of gnotobiotic mice and monozygotic twins. Sci. Transl. Med..

[24] Halket JM (1999). Deconvolution gas chromatography/mass spectrometry of urinary organic acids–potential for pattern recognition and automated identification of metabolic disorders. Rapid Commun. Mass Spec..

[25] Kind T (2009). FiehnLib: mass spectral and retention index libraries for metabolomics based on quadrupole and time-of-flight gas chromatography/mass spectrometry. Anal. Chem..

[26] Kopka J (2005). GMD@CSB.DB: the Golm Metabolome Database. Bioinformatics.

[27] Breiman, L., Friedman, J., Olshen, R. & Stone, C. Classification and Regression Trees. CRC Press, Boca Raton, FL (1984).

[28] Matlab R. Statistics and Machine Learning Toolbox, classification trees (fitctree). (2018).

