# Supplementary material for: Non-targeted urinary metabolomics in pregnancy and associations with fetal growth restriction
Source: Sci Rep. 2020 Mar 24;10:5307. doi: 10.1038/s41598-020-62131-7 (PMC7093500; doi:10.1038/s41598-020-62131-7)
Supplement: Supplementary file 1 — Supplementary Materials. [file 41598_2020_62131_MOESM1_ESM.pdf]

Non-targeted urinary metabolomics in pregnancy and associations with fetal growth restriction

Chelsea M. CLINTON, M.D., James R. BAIN, Ph.D., Michael J. MUEHLBAUER, Ph.D., YuanYuan LI, Ph.D., Leping LI, Ph.D., Sara K. O'NEAL, M.S., Brenna L. HUGHES, M.D., David E. CANTONWINE, Ph.D., Thomas F. MCELRATH, M.D., Ph.D., Kelly K. FERGUSON, Ph.D.

Supplemental Materials

Supplemental methods. CART analysis and validation.

Supplemental Figure 1. Average classification accuracies from 60 training-testing partitions as a function of the tree size (the minimum number of samples in each leaf node for Visit 1 (A) and Visit 3 (B). The vertical dashed lines indicate the best training-testing trade-offs.

Supplemental Table 1. Urinary metabolite percent below detection limit by case status and visit number for 137 analytes included in the present study.

Supplemental methods: CART analysis and validation

Through cross-validation, we found that the loss function based on the Gini's diversity index (GDI) gave the best performance. GDI measures the class diversity in the nodes of a decision tree. In binary classification trees, Gini index prefers splits that put the largest class into one pure node, and all others into the other. This splitting criterion is used, for example, in CART. The GDI of a node is defined as $1 - \sum_i p^2(i)$, where the sum is over the classes i at the node, and $p(i)$ is the observed fraction of classes with class i that reach the node. A node with just one class (a pure node) has Gini index 0; otherwise the Gini index is positive. Therefore, the Gini index is a measure of node impurity.

Fitctree uses tree size to control "bias-variance" trade-offs. Large trees may fit the training data well but may fit the testing data poorly, a situation called "overfitting". To avoid overfitting, one could adjust model complexity by the changing values of tuning parameters: maximum number of splits, or minimum number of observations in leaf. The number of splits and the number of observations in leaf determine the final tree's structure and complexity. Since both parameters control the tree size and the minimum number of leaf node observations gives us finer control over tree structures, we only tuned the values of the minimum number of leaf node observations.

To identify the best tree structure for the metabolite dataset, we carried out a leave-one-out cross-validation procedure on the data. In leave-one-out cross-validation, one sample was sequentially set aside as the testing sample and the remaining samples were used as the training samples. This procedure created 60 training-testing partitions of the original dataset. We sequentially limited the minimum number of samples in a leaf node from 1 to 30 (half of the sample size) to test the effect of the tree size on classification accuracy. For each training set, we

fit a classification tree and the resulting tree was used to classify the corresponding sample in the testing set. For the 60 testing sets, we counted the number of times the testing sample was corrected classified. The average classification accuracies as a function of tree size for the training and testing sets are shown in Supplemental Figure 1. Based on the best training-testing performance trade-offs, the average training and testing accuracies are 83.1% and 73.3%, respectively for Visit 1 and 81.4% and 76.3%, respectively for Visit 3.

Supplemental Figure 1. Average classification accuracies from 60 training-testing partitions as a function of the tree size (the minimum number of samples in each leaf node for Visit 1 (A) and Visit 3 (B). The vertical dashed lines indicate the best training-testing trade-offs.

Supplemental Figure 1a.

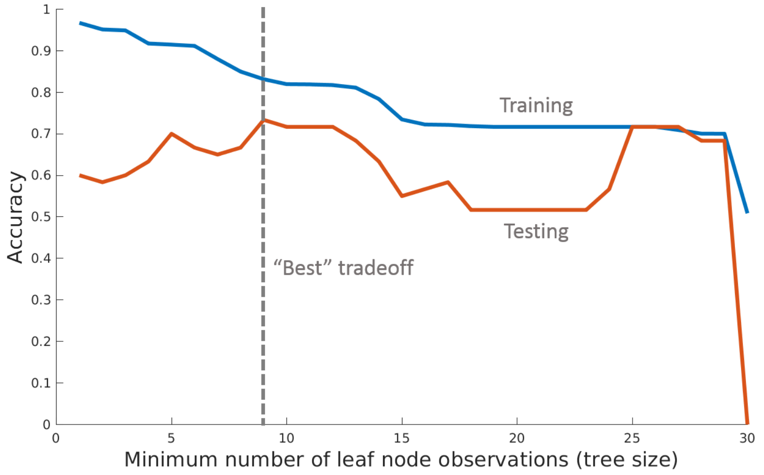

Supplemental Figure 1b.

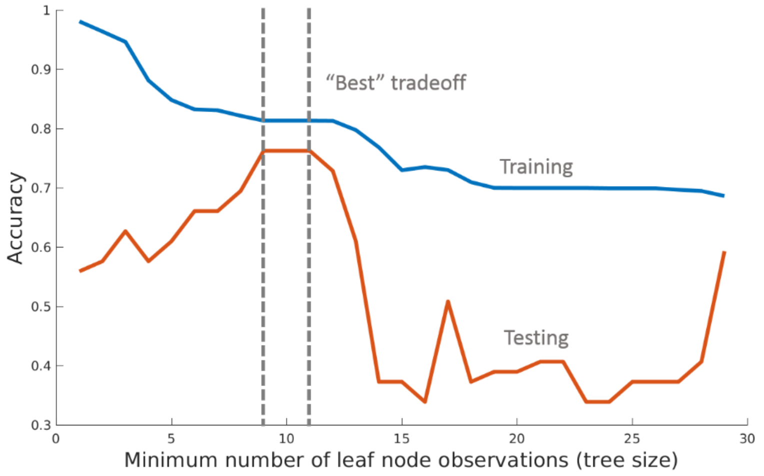

Supplemental Table 1. Urinary metabolite percent below detection limit by case status and visit number for 137 analytes included in the present study.

	Cases		Controls	
**Urinary metabolite**	**Visit 1**	**Visit 3**	**Visit 1**	**Visit 3**
1,2-Propanediol	0.00	0.00	0.00	0.00
Acetoacetate	0.00	0.00	0.00	0.00
3-Methylcrotonylglycine	6.72	10.92	8.40	8.40
2-Hydroxyphenylacetic acid	0.00	0.00	0.00	0.00
alpha Ketoglutaric acid	0.00	0.00	0.00	0.00
3-Hydroxy-3-methylglutaric acid (dicrotalic acid)	0.84	0.00	0.84	0.84
Pimelic acid	0.00	0.00	0.00	0.00
3-Hydroxyphenylacetic acid	0.00	0.00	0.00	0.00
2-Hydroxybutyric acid	5.88	5.88	10.92	8.40
Anthranilic acid	0.00	0.00	0.00	0.00
Phenylalanine	0.00	0.00	1.68	0.84
4-Hydroxybenzoic acid	0.00	0.00	0.00	0.00
4-Hydroxyphenylacetic acid	0.00	0.00	0.00	0.00
2,5-Furandicarboxylic acid	0.00	0.00	0.84	0.00
Lauric acid	0.84	0.84	0.00	2.52
N-2-Furoylglycine	1.68	0.00	2.52	0.00
Oxalic acid	0.84	0.00	0.84	0.00
N-Acetylaspartic acid	0.84	0.00	0.00	0.00
2-Hydroxyhexanedioic acid	4.20	2.52	3.36	2.52
Ketopentoses	10.08	3.36	5.88	5.88
Aldopentoses	15.13	5.04	12.61	10.92
Phthalic acid	12.61	10.92	8.40	15.97
N-Acetylcysteine	15.13	9.24	15.97	9.24
Suberic acid	0.00	0.00	0.00	0.00
Quinolinic acid	7.56	3.36	9.24	4.20
3-(3-Hydroxyphenyl)propionic acid	3.36	2.52	4.20	0.00
2-Hydroxy-2-methylbutyric acid	1.68	0.84	0.84	0.00
Pentitols	0.00	0.00	0.00	0.00
Orotic acid	5.88	7.56	8.40	8.40
Aconitic acid	0.00	0.00	0.00	0.00
N-Acetylglutamic acid	5.04	0.84	0.00	0.84
Gentisic acid	4.20	4.20	1.68	3.36
4-Hydroxy-3-methoxybenzoic acid	1.68	1.68	0.84	0.00
Homovanillic acid	0.00	0.00	0.00	0.00
4-Hydroxymandelic acid	0.00	0.00	0.00	0.00

Azelaic acid	0.00	0.00	0.00	0.00
Hippuric acid	0.00	0.00	0.00	0.00
Citric acid/isocitric acid	0.00	0.00	0.00	0.00
3,4-Dihydroxybenzoic acid	1.68	5.04	1.68	5.04
Saccharin	15.13	11.76	9.24	12.61
3,4-Dihydroxyphenylacetic acid	7.56	10.08	11.76	7.56
2-Methylcitric acid	0.00	0.00	0.00	0.84
Myristic acid or Pentadecanol	7.56	14.29	10.92	12.61
Vanillylmandelic acid	0.00	0.00	0.00	0.00
trans-3-Hydroxycinnamic acid	10.92	6.72	10.08	6.72
Hydantoin-5-propionic acid (or similar histidine metabolite)	0.00	5.04	0.84	5.88
p-Hydroxyphenyllactic acid	0.00	0.00	0.00	0.00
Urocanic acid	2.52	5.88	5.04	2.52
4-Hydroxyphenylpyruvic acid	1.68	0.84	0.84	0.00
p-Cresol	0.00	0.00	0.00	0.00
Dehydroascorbic acid	9.24	13.45	10.92	13.45
Hexuronic acid	15.13	9.24	14.29	10.92
Tyrosine	6.72	10.92	12.61	10.08
4-Hydroxycinnamic acid	5.88	6.72	8.40	5.04
3-Indoleacetic acid	0.00	0.00	0.00	0.84
3-Carboxy-4-methyl-5-propyl-2-furanpropanoic acid (CMPF)	10.92	10.92	11.76	10.08
Pantothenic acid	0.00	0.00	0.00	0.00
1-Hydroxy-2-naphthoic acid	5.88	4.20	4.20	5.04
Palmitic acid	0.00	0.00	0.00	0.00
Kynurenic acid	0.84	11.76	5.04	5.88
trans-Ferulic acid	0.84	1.68	2.52	0.00
Uric acid	2.52	9.24	1.68	5.04
Indole-3-acetamide	0.00	9.24	1.68	5.04
N-Acetyltyrosine	3.36	4.20	5.88	3.36
p-Hydroxyhippuric acid	0.00	0.00	0.00	0.00
2-Hydroxyvaleric acid	0.00	0.00	0.00	0.00
3-Indolelactic acid	10.92	5.04	15.97	8.40
Cinnamoylglycine	1.68	0.84	3.36	1.68
5-Hydroxyindole-3-acetic acid	0.84	0.00	0.84	1.68
Tryptophan	10.08	16.81	6.72	15.13
Oleic acid	1.68	0.84	1.68	0.84
Stearic acid	0.00	0.00	0.00	0.00
n-Heptanoic acid	0.00	0.84	0.00	1.68
Xanthurenic acid	0.00	5.04	0.00	1.68

N-Acetyltryptophan	5.04	5.04	5.04	5.88
Uridine	3.36	4.20	6.72	5.88
alpha-Monopalmitin	0.00	2.52	1.68	1.68
alpha-Monostearin	0.84	0.00	0.00	0.84
Pyruvic acid	0.00	0.00	0.00	0.00
Estriol	11.76	2.52	15.97	4.20
Cholesterol	0.00	0.00	0.00	0.00
2-Ketoleucine/ketoisoleucine	0.00	0.00	0.00	0.00
Succinic semialdehyde	0.84	0.00	0.00	0.84
Methylmalonic acid	0.84	0.00	0.00	0.00
Glyceraldehyde	8.40	4.20	5.04	4.20
Malonic acid	0.00	0.00	0.84	0.00
Guaiacol	11.76	7.56	9.24	7.56
gamma-Hydroxybutyric acid	3.36	3.36	5.04	0.84
Urea	0.00	0.00	0.00	0.00
Benzoic acid	0.00	0.00	0.00	0.00
Phenol	0.00	0.00	0.00	0.00
Oxamic acid	0.84	0.00	0.84	0.84
Octanoic acid	0.00	0.00	0.84	0.00
Phosphoric acid	0.00	0.00	0.00	0.00
Ethylmalonic acid	0.00	0.00	0.00	0.00
4-Vinylphenol	9.24	3.36	8.40	5.04
2-Phenylacetic acid	3.36	4.20	1.68	2.52
Glycine	7.56	3.36	1.68	4.20
Lactic acid	0.00	0.00	0.00	0.00
Succinic acid	0.00	0.00	0.00	0.00
Catechol	6.72	5.04	5.04	2.52
Methylsuccinic acid	0.00	0.00	0.00	0.00
Glyceric acid	1.68	2.52	2.52	0.84
Uracil	0.00	0.00	0.00	0.00
Citraconic acid	1.68	0.84	1.68	0.84
Fumaric acid	1.68	0.84	0.00	0.84
2-Hydroxyisobutyric acid	0.00	0.00	0.00	0.00
Nonanoic acid	0.00	0.00	0.00	0.00
Threonic acid-1,4-lactone	11.76	9.24	12.61	6.72
Tartronic acid	9.24	10.08	10.92	9.24
Mesaconic acid	0.00	0.84	0.00	0.00
Hydroquinone	8.40	9.24	10.92	6.72
Glutaric acid	0.00	0.00	0.00	0.00
2-Methylglutaric acid	2.52	5.88	8.40	3.36

Isobutyrylglycine	0.00	3.36	2.52	2.52
3-Methylglutaric acid	0.00	0.00	0.00	0.00
Glycolic acid	0.00	0.00	0.00	0.00
Benzamide	8.40	4.20	7.56	2.52
3-Methylglutaconic acid	0.00	0.00	0.00	0.00
Indole	12.61	3.36	10.92	4.20
Decanoic acid	0.84	0.84	1.68	0.84
2-Phenylacetamide	5.04	2.52	2.52	2.52
2-Ketobutyric acid	5.88	15.13	15.97	10.08
Citramalic acid	0.00	0.00	0.00	0.00
Mandelic acid	1.68	0.00	1.68	0.84
Malic acid	4.20	0.84	2.52	1.68
2-Methylbutyrylglycine	5.04	8.40	4.20	5.88
4-Acetamidobutyric acid	9.24	7.56	10.08	3.36
Adipic acid	0.00	0.00	0.00	0.00
Isovalerylglycine	0.00	1.68	0.84	1.68
Acetylsalicylic acid/Salicylic acid	0.00	2.52	1.68	0.84
2-Ketovaline	0.84	1.68	0.84	0.00
Glutamic acid	0.00	0.00	0.00	0.00
Erythronic acid	0.00	0.84	0.84	1.68
5-Hydroxymethyl-2-furoic acid (Sumiki's acid)	0.84	0.84	0.84	0.00
Tiglyl glycine	2.52	5.88	3.36	3.36
Threonic acid	0.00	0.00	0.00	0.00
